# Renal abnormalities among HIV infected children at Muhimbili National Hospital (MNH)—Dar es Salaam, Tanzania

**DOI:** 10.1186/s12882-016-0242-6

**Published:** 2016-03-21

**Authors:** Francis Fredrick, Joel M. Francis, Paschal J. Ruggajo, Eden E. Maro

**Affiliations:** Department of Paediatrics and Child Health, School of Medicine, Muhimbili University of Health and Allied Sciences (MUHAS), P. O. Box 65001, Dar es Salaam, Tanzania; Renal Unit, Muhimbili National Hospital (MNH), Dar es Salaam, Tanzania; National Institute of Medical Research (NIMR), Mwanza-Centre, Mwanza, Tanzania; Department of Internal Medicine, School of Medicine, Muhimbili University of Health and Allied Sciences (MUHAS), Dar es Salaam, Tanzania

## Abstract

**Background:**

Human Immunodeficiency Virus infection is a multisystem disease that contributes to significant morbidity. Renal involvement is reported to be common among patients with HIV. This study was carried out to determine renal involvement using simple bedside tests combined with ultrasonography examination.

**Methods:**

We recruited 240 children from the HIV clinic at Muhimbili National Hospital. Data were collected using structured questionnaires and included demographic, clinical information, radiological tests; renal ultrasound and laboratory tests; serum creatinine, white blood cells, CD4+ counts and percent, urine for microalbuminuria and proteinuria.

**Results:**

Microalbuminuria and proteinuria were present in 20.4 % and 7.1 % respectively. Significantly higher prevalence of microalbuminuria (*p* < 0.01) and proteinuria *p* < 0.01) were noted with low CD4 percent (<25 %). Lower mean CD4+ count were noted among children with microalbuminuria [937.4 ± 595.3 cells/μL vs 1164.7 ± 664.3 cell/μL, (*p* < 0.05)] and proteinuria [675.5 ± 352.3 cells/μL vs 1152 ± 662 cells/μL (*p* < 0.001)]. Fourteen (5.8 %) HIV infected children had estimated glomerular filtration rate (eGFR of 30–59) consistent with severe renal impairment. Increased cortical echogenicity was noted in 69/153 (39.2 %) of participants who had ultrasound examination.

**Conclusion:**

Microalbuminuria, proteinuria and renal dysfunction were noted to be prevalent among HIV infected children indicating the need to consider routine screening of renal complications in these children.

## Background

About 2.3 million children under the age of 15 years were estimated to be living with HIV globally 90 % of which were in sub-Saharan Africa [1]. In Tanzania 150,000 children aged between 0 and 14 years were estimated to require anti-retroviral therapy (ART) in 2011 [2]. Deaths in the first two years of life were attributed mainly to diarrhoeal diseases and respiratory infections before ART programs. [3] Improved survival with ART programs has paved way for long term HIV complications including renal complications [4]. ART drugs have been reported to contribute to renal complications [5].

Dondo et al. reported renal impairment in 34.6 % and proteinuria in 16 % among ART naïve HIV infected children in Zimbabwe [6]. In Nigeria proteinuria was reported in 20.8 % of HIV infected children by Esezobor et al. [7] In a study of ten children with HIV nephropathy in Nigeria Anochie et al. described proteinuria in all participants and impaired renal function in 90 % [8]. These findings highlight the contribution of renal complications in morbidity and possible mortality in HIV infected African children.

HIV related complications in HIV infected children include proteinuria, electrolyte disturbances, urinary tract infections, impaired renal function and end stage renal disease (ESRD) [9, 10]. Proteinuria is a marker of HIV nephropathy and has been correlated with decreased renal function and progression to ESRD. [11–13] HIV infected patients with advanced AIDS stage, severe malnutrition, low CD4 count and high viral load have been reported to be more likely to present with proteinuria [5, 6, 13, 14].

Despite proteinuria being the early marker of HIV nephropathy, microalbuminuria has been reported to predict its occurrence [15]. In a study which was conducted in Bethesda, USA in a cohort of HIV infected children 15 % were noted to have microalbuminuria which correlated significantly with low CD4 count [16]. HIV infected patients with microalbuminuria have been reported to have kidney abnormalities, Han et al reported biopsy proven HIV nephropathy in majority (86 %) of HIV infected patients who had microalbuminuria in South Africa [11].

HIV renal complications results from direct infection of renal epithelial and mesangial cells and deposition of immune complexes into renal tissues [17, 18]. Anti-retroviral medications have also been reported to cause renal complications [19–21]. This study aimed to determine renal complications and associated factors among HIV infected children attending HIV clinic at Muhimbili National Hospital (MNH).

## Methods

This was a hospital based cross-sectional study of HIV infected children attending HIV clinic at MNH. Muhimbili University of Health and Allied Sciences Research Ethical Committee approved this study. We obtained informed written consents from parents/guardians and assent from participants aged ≥10 years prior to recruitment.

HIV infected children aged 1–14 years were consecutively recruited between January and March, 2011. We estimated a minimum sample size of 250 to determine proteinuria using *Epi info version 6* statistical package assuming prevalence of microalbuminuria of 15 % based on the findings of a study conducted in a cohort of children infected with HIV acquired vertically in Bethesda [16].

Standardized questionnaires were used to obtain information on socio-demographic information including age and sex of participants. Other information was obtained from case notes. Clean catch urine specimen from each participant was tested for proteinuria and microalbuminuria using URiSCAN® dipstick and Microalbumin 2-1 test strips (which determine albumin to creatinine ratio) respectively. Proteinuria was defined as positive dipstick test of ≥ +1 corresponding to ≥ 30 mg/dL and microalbuminuria was defined as urinary albumin/creatinine ratio of ≥30 mg/g. Five milliliters of blood samples were drawn from each participant and tested for CD4 count and percentage, white blood cell count and serum creatinine using Architect Chemistry analyzer (Abbot®) and Cell-dyn 3500R analyzer respectively.

Each participant had weight measurement taken using a calibrated standard beam balance (SECA®) with the child putting on light clothing and no shoes, weight measures were approximated to the nearest 10 g. We calibrated the weighing scale to zero each day of recruitment. Length of children was measured with a length board, parents/guardians assisted in removing shoes and gently laying the child in supine position on the board, with their heads placed at 90° to the fixed headpiece, the sliding foot piece was brought into contact to the child’s heels. Height of participants was measured with the child standing at right angle in front of height board and sliding head piece was brought into contact with the head. Height/length was recorded to the nearest 10 mm. Body Mass Index (BMI) was calculated for each participant and was used to determine nutrition status using BMI for age z-score according to World Health Organization (WHO) standard charts.

Estimated GFR (eGFR) was calculated using modified bedside Schwartz equation validated for children with and without CKD [22–25]. Recruited participants were given appointments for renal ultrasound examination scheduled one week after recruitment. Two certified radiologists reviewed the images and reached an agreement. Renal size was determined by comparison with age matched norms. Cortical echogenicity of the sonograms were graded relative to the liver or spleen. Cortical echogenicity was compared with liver and spleen and children who had higher echogenicity as compared to liver and spleen were considered to have abnormally increased echogenicity.

Stata version 12 was used for data analysis, proportions were calculated for microalbuminuria, proteinuria, *t*-test was used to compare continuous variables while association between categorical variables was determined using Chi-square and Fisher’s exact tests. Univariable logistic regression and multivariable logistic models were performed to determine predictors of microalbuminuria and proteinuria. Crude and adjusted odds ratio (OR) were reported, p value of < 0.05 was considered statistically significant.

## Results

### Demographic and clinical characteristics of study participants

Two hundred and forty children were recruited into this study as described in Table 1, out of which 133 (55.4 %) were males. Participants were aged between 1 and 14 years with mean age of 7.6 ± 3.7 years. Majority of study participants 219 (91.2 %) were using anti-retroviral drugs (ARV), of those using ARV 53 % (129/219) were using stavudine, lamivudine and nevirapine combination regimen. Sixty four (27.6 %) participants had immunosuppression (CD4 < 25 %), 44 (20.4 %) participants had microalbuminuria and 17 (7.1 %) had proteinuria.

### Glomerular filtration rate and renal ultrasound findings

Fourteen (5.8 %) participants had eGFR between 30 and 59 ml/min/m2 which is consistent with renal impairment, 83 (34.6 %) had eGFR of ≥ 90 ml/min/m2 and 143 (59.6 %) participants had eGFR between 60 and 89 ml/min/m2. All participants were given appointments for renal ultrasound examination but only 153 (63.8 %) out of all participants attended for this evaluation. Sixty participants (39.2 %) out of 153 who were examined had normal kidney size and increased echogenicity.

### Factors associated with microalbuminuria

Presence of haematuria and low CD4 percent were noted to influence occurrence of microalbuminuria. We observed that children with lower CD4 percent (<25 %) to more likely to have microalbuminuria [39.5 % vs. 14.8 % (*p* < 0.01)] as compared to those with CD4 percent ≥ 25 %, similarly higher proportion of participants with microalbuminuria had haematuria as compared to those without [50.0 % vs. 19.0 % (*p* = 0.02)] Table 2.

**Table 1:** Demographic characteristics of study participants

Variable	N (%)/N (±SD)
Age	
< 5 years	66 (27.5)
5 up to 10 years	94 (39.2)
≥ 10 years	80 (33.3)
Sex	
Male	133 (55.4)
Female	107 (44.6)
Using anti-retroviral therapy	
Yes	219 (91.2)
No	21 (8.8)
Types of ARV	
d4T, 3TC, NVP	129 (53.8)
d4T, 3TC, EFV	33 (13.8)
AZT, 3TC, NVP	28 (11.7)
AZT, 3TC, EFV	24 (10.0)
ABC, ddI, Kaletra	5 (2.1)
CD4 percent	
≥ 25 %	64 (27.6)
< 25 %	176 (73.3)
Laboratory values	
Mean serum creatinine	51.3 ± 6.8 μmol/L
Mean blood urea nitrogen	2.8 ± 0.9 mmol/L
Mean eGFR	84 ± 14.7 mL/min/1.73 m^2^
Mean white blood cell count	6.7 ± 2.8 x 10^3^ cells/μL
Mean CD4 percent	31.1 ± 10 %
Mean haemoglobin level	10.9 ± 1.4 g/dL

**Table 2:** Factors associated with microalbuminuria

Variable	n	Microalbuminuria present n (%)	*P* value
Age (years)			
1 up to 5	63	12 (18.2)	
5 up to 10	91	18 (19.1)	
10 and above	86	19 (23. 8)	0.669
Sex			
Male	133	30 (22.6)	
Female	107	19 (17.8)	0.359
Use of ART			
Yes	219	45 (20.5)	
No	21	4 (19.0)	1.000
CD4 percent			
< 25 %	64	23 (35.9)	
≥ 25 %	176	26 (14.8)	0.001
Haematuria			
Present	10	5 (50.0)	
Absent	230	44 (19.1)	0.018
BMI z–score			
Median- -1SD	164	39 (23.8)	
< -1 SD	76	10 (13.2)	0.061
Renal echogenicity^a^			
Normal	93	14 (23.3)	
Increased	60	20 (21.5)	0.843

^a^ *n* = 153

**Table 3:** Factors associated with Proteinuria (*n* = 240)

Variable	n	Proteinuria present n (%)	*P* value
Age (years)			
1 up to 5	63	4 (6.1)	
5 up to 10	91	2 (2.1)	
10 and above	86	11 (13.8)	0.012
Sex			
Male	133	9 (6.8)	
Female	107	8 (7.5)	0.831
Use of ART			
Yes	119	16 (7.3)	
No	21	1 (4.8)	1.000
CD4 percent			
< 25 %	64	10 (15.6)	
≥ 25 %	176	7 (4.0)	0.004
Haematuria			
Present	10	1 (10.0)	
Absent	230	16 (7.0)	0.527^a^
BMI Z –score			
Median- -1SD	164	11 (6.7)	
< -1 SD	76	6 (7.9)	0.739
Renal echogenicity^b^			
Normal	93	6 (10.0)	
Increased	60	9 (9.7)	1.0

^a^Fischer’s exact test, ^b^ *n* = 153

Children with haematuria had four folds increased likelihood of having microalbuminuria as compared to those without haematuria. Children with low CD4 percent (<25 %) had five fold increased likelihood of having microalbuminuria as compared to those with CD4 ≥ 25 %, Table 4.

**Table 4:** Univariate and multivariate logistic regression for factors associated with microalbuminuria

Variable	Crude OR (95 % CI)	*p*-value	Adjusted OR (95 % CI)	*p*-value
Age				
< 5 years	Ref		Ref	
5–10 years	1.166 (0.508–2.673)	0.717	2.242 (2.396–11.124)	0.116
> 10 years	1.432 (0.630–3.254)	0.391	3.020 (1.0345–8.820)	0.043
Sex				
Male	Ref		Ref	
Female	0.741 (0.390–1.407)	0.360	0 .656 (0.309–1.393)	0.273
CD4 percent				
≥ 25 %	Ref		Ref	
< 25 %	3.236 (1.675–6.254)	<0.0001	5.134 (2.369–11.125)	<0.0001
Haematuria				
Absent	Ref		Ref	
Present	4.227 (1.172–15.240)	0.028	3.844 (0.975–15.153)	0.054

### Factors associated with proteinuria

The proportion of children with proteinuria was higher among children aged ten years and above as compared to younger children (*p* = 0.01). Children with lower CD4 percent (< 25 %) were likely to present with proteinuria [15.6 % vs. 4.0 % (*p* < 0.01)] as compared to those with CD4 percent ≥ 25 %, Table 3. Participants with proteinuria had significantly lower mean CD4 count as compared to those without proteinuria [675.5 ± 352.3 cell/μL vs. 1152 ± 662 cells/μL (*p* < 0.01)]. Participants with CD4 < 25 % were four fold more likely to have proteinuria as compared to those with CD4 ≥ 25 %, Table 5. No association was noted between haematuria or severe renal impairment with microalbuminuria or proteinuria.

**Table 5:** Univariate and multivariate logistic regression for factors associated with proteinuria

Variable	Crude OR (95 % CI)	*p*-value	Adjusted OR (95 % CI)	*p*-value
Age				
< 5 years	Ref		Ref	
5–10 years	0.331(0.059–1.868)	0.211	0.539(0.081–3.593)	0.523
> 10 years	2.163(0.655–7.140)	0.205	3.538(0.728–17.190)	0.117
CD4 percent				
< 25 %	Ref		Ref	
≥ 25 %	4.471(1.623–12.316)	<0.004	4.416(1.409–13.845)	0.011
Haematuria				
Present	Ref		Ref	
Absent	1.486(0.177–12.474)	0.715	0.978(0.104–9.175)	0.984

## Discussion

We found microalbuminuria and proteinuria to be common among HIV infected children in our setting. Microalbuminuria was noted in 20.4 % while proteinuria was present in 7.1 % of the recruited participants in this study. This findings were presumed to represent HIVAN.

Microalbuminuria has been reported as a common renal manifestation in HIV infected people, several studies have reported microalbuminuria in HIV infected children both in developed countries and sub-Saharan Africa. Dimock et al reported 15 % among HIV infected youth and children in Bethesda, USA [16]. Two other studies conducted among HIV infected Nigerian children reported prevalence of microalbuminuria of 11.1 % and 12 % [26, 27]. Microalbuminuria present earlier than proteinuria and has been reported to predict proteinuria in HIV infected patients [15].

A study conducted among HIV infected Zimbabwean children reported a prevalence of proteinuria to be 5 %, this is comparable to our findings of 7.1 % [6]. Esezobor et al. [7] reported a prevalence of proteinuria determined using urine protein to creatinine ratio, to be 20.5 % in a study conducted among HIV infected Nigerian children [7].

Older children had significantly higher prevalence of proteinuria in our study; with no difference being noted in occurrence of microalbuminuria with age. Therefore it may be advantageous to screen children with both tests in order to detect earlier renal complications. Microalbuminuria has been reported as a presentation of HIV associated nephropathy, in South Africa Han et al reported microalbuminuria in 24 % of patients who had biopsy proven HIV associated nephropathy [11].

Participants with immunosuppression (CD4 percent < 25 %) had higher prevalence of both microalbuminuria and proteinuria in this study. This is consistent with reports from other studies, Esezobor et al. in a study conducted in Nigerian children reported proteinuria in 11 (37.9 %) out of 29 children who had severe immunosuppression as compared to 7 (11.9 %) out of 59 with milder immunosuppression [4]. Chaparro et al reported similar association between proteinuria and low CD4 count and high viral load in a landmark study of proteinuria in HIV infected children conducted in Miami, USA [13].

Participants with microalbuminuria and proteinuria had significantly lower mean CD4 count which is in consistence with noted trend for CD4 percent. Similar findings have been reported among HIV infected patients with immunosuppression and advanced disease [6, 7, 13–15, 26, 27]. It is evident from our findings that it is important for clinician to make close follow up on the progress of patients receiving ART, to ensure immune competency is attained and maintained to prevent or delay occurrence of HIV associated renal complications. Fifteen participants (0.06 %) had haematuria which had no significant association with mircroalbuminuria or proteinuria; this finding may be attributed to urinary tract infection.

Severe renal impairment (defined as eGFR <60 ml/min/1.73 m^2^) was noted in 5.8 % of study participants, this is lower than 13.3 % reported by Esezobor et al. from a study conducted among HIV infected children in Nigeria [28]. In our study eGFR was estimated using serum creatinine which is different from cystatin C method utilized by Esezobor et al. Dondo et al in study conducted among HIV infected children in Zimbabwe reported renal dysfunction (defined by eGFR of 30 to ≤ 90 ml/min/1.73 m^2^) in 34.6 % of participants [6]. These findings justify initial renal function screening at recruitment and regular monitoring in HIV infected children.

Renal ultrasound examination of 153 participants revealed normal sized kidneys with increased cortical echogenicity in 39.2 % (60/153) which was attributed to HIVAN. Similar findings were reported by Steel-Duncan et al. in a study which was conducted in HIV infected Jamaican children which revealed normal sized kidneys with increased echogenicity [29]. Anochie et al reported grossly enlarged kidneys in 30 % and normal sized kidneys and increased echogenicity in 70 % of children with HIVAN in Nigeria [8]. No correlation was noted between occurrence of microalbuminuria or proteinuria and increased echogenicity in our study. It may be important to performing renal ultrasound examination at recruitment and regularly thereafter to complement urinalysis and renal function tests.

## Study limitations

Microalbuminuria and proteinuria were tested only once in this study this could have underestimated the true magnitude of these two entities. The modified Schwartz formula utilized for estimating eGFR could have resulted in over/underestimation of GFR in the study population particularly for children below two years. Only 63.8 % of participants had renal ultrasound examination performed, this is because participants were given appointment for the examination on a different day from the visit on which they were recruited, this may be due to lack of reimbursement for transport which could not be provided in this study.

## Conclusions

This study has documented existence of renal abnormalities in HIV infected children at MNH in the form of microalbuminuria, proteinuria and renal dysfunction. Immunosuppression was positively linked to occurrence of both microalbuminuria and proteinuria indicating the importance of attaining immune-competency among HIV infected children who are ART naïve and those on ART. Findings of this study may justify the role of screening HIV infected children for renal complications before starting ART and annually thereafter. This will facilitate earlier detection of affected children and making proper follow up including seeking care from kidney care specialists.

## Abbreviations

AIDS: acquired immunodeficiency syndrome
ART: anti-retroviral therapy
BMI: body mass index
CD: cluster of differentiation
CKD: chronic kidney disease
eGFR: estimated glomerular filtration rate
ESRD: end stage renal disease
HIV: human immunodeficiency virus
HIVAN: human immunodeficiency virus associated nephropathy
MNH: Muhimbili National Hospital
MUHAS: Muhimbili University of Health and Allied Sciences
NIMR: National Institute of Medical Research
OR: odds ratio
USA: United States of America
WHO: World Health Organization
